# Effect of Microwave Radiation on Enzymatic and Chemical Peptide Bond Synthesis on Solid Phase

**DOI:** 10.1155/2009/362482

**Published:** 2009-03-25

**Authors:** Alessandra Basso, Loris Sinigoi, Lucia Gardossi, Sabine Flitsch

**Affiliations:** ^1^Laboratory of Applied and Computational Biocatalysis, Department of Pharmaceutical Sciences, University of Trieste, Piazzale Europa 1, 34127 Trieste, Italy; ^2^School of Chemistry, The University of Manchester, 131 Princess Street, Manchester M1 7DN, UK; ^3^Manchester Interdisciplinary Biocentre (MIB), The University of Manchester, 131 Princess Street, Manchester M1 7DN, UK

## Abstract

Peptide bond synthesis was performed on PEGA beads under microwave radiations. Classical chemical coupling as well as thermolysin catalyzed synthesis was studied, and the effect of microwave radiations on reaction kinetics, beads' integrity, and enzyme activity was assessed. Results demonstrate that microwave radiations can be profitably exploited to improve reaction kinetics in solid phase peptide synthesis when both chemical and biocatalytic strategies are used.

The use of microwave (MW) heating has found successful applications in solid phase organic synthesis (SPOS) [1–7].

As far back as 1992, Wang described the use of a single-mode microwave as a heating source to accelerate the chemical coupling [8] of many amino acids. Reactions on SP often suffer from unsatisfactory reaction kinetics due to slow diffusion. Since microwave energy activates any molecule with dipole moment, a rapid heating at a molecular level is achieved, along with an improved diffusion of solutes.

As an alternative to chemical coupling strategies, peptide bond can be synthesized also via protease-catalyzed reactions [9–11]. Proteases catalyze the thermodynamically controlled synthesis in aqueous buffer while avoiding racemization and the needs for side chain protection of amino acids [12].

Thanks to their small molecular weight, proteases, such as thermolysin (35 kDa), diffuse efficiently in a range of different swelling and rigid polymers, such that high conversions can be achieved especially when hydrophobic amino acids are used. Moreover, it was observed that the formation of the peptide bond is thermodynamically favored when performed on SP—as compared to the process carried out in solution—even though the heterogeneous reaction takes place in a bulk aqueous environment [12].

In this paper, we report the effect of microwaves in the formation of peptide bond on SP, which also represents the first example of MW application in solid phase biocatalysis.

The swelling PEGA polymer (cross-linked acrylamide and polyethylene glycol) was chosen as solid support since it was previously found to be completely permeable to proteins with molecular masses up to 35 kDa [9–15].

As depicted in Scheme 1, the dipeptide Fmoc-Phe-Phe was synthesized using an MW assisted procedure starting from PEGA-Phe, which was chemically synthesized.

The biocatalyzed reaction was carried out using a thermostable protease, thermolysin (Thermolysin from *Bacillus thermoproteolyticus rokko*) which had been previously employed for peptide synthesis on PEGA beads [9].

As a comparison, MW assisted chemical coupling was carried out using the traditional HOBt/DIC approach, which has been previously employed in the MW assisted peptide synthesis in solution [16].

The MW assisted enzymatic synthesis of the dipeptide Fmoc-Phe-Phe-PEGA_1900_, Scheme 1, was monitored for a range of temperatures from 50°C to 100°C at a power of 50 W (see Supplementary Material). The reactions were followed by determining the amount of Fmoc on the resin (Figure 1) [15]. Product was also analyzed by HPLC after cleavage from the resin and verifying its chemical purity and enantiopurity.

Figure 2 reports the initial rates [15] of the MW assisted enzymatic synthesis as function of the temperature.

It appears that at 80 and 90°C, the highest enzymatic activity can be achieved. However, from the reaction profiles reported in Figure 2 it is evident that only at 80°C the enzymatic coupling was complete after one hour whereas at 90 and 100°C the enzyme undergoes a fast denaturation (bold and empty squares) so that complete conversion was achieved only upon addition of fresh thermolysin. It is interesting to note that similar profiles had been previously reported for a different enzyme, the lipase B from *Candida antarctica*, that showed the highest activity at 60° C [17, 18].

The graph reported in Figure 1 also shows that at temperature ranging from 50 to 70°C the reaction achieves completion within 3 to 4 hours.

A nonthermal effect due to microwaves is evident when comparing the initial rates measured at 50 and 80°C in the absence of MW (in a thermostated bath, Figure 1, bold circles and empty triangles, respectively); at 80°C the initial rate is eight times lower.

Similar nonthermal effects have been reported for protease esterification and transesterification [19], whereas in lipase-catalyzed reactions no microwave effect was observed [19–23].

For comparison, the chemical coupling on SP was also carried out (Figure 3). Complete conversion was obtained at 50°C and 50 W power after 30 minutes [13]. (All the amino groups, initially present on the resin (0.15 mmol/g_dry_), were acylated as confirmed by loading determination.) It must be underlined that the same chemical coupling in the absence of MW radiation at the same temperature requires two reaction cycles, one of 2 hours and one of 24 hours, respectively [14, 15].

The swelling capacity of PEGA_1900_ in water was not affected by microwave heating so that after 30 minutes incubation in water at 100°C under microwave radiation the determined swelling capacity remained 23 mL/g, as in the case of an identical sample incubated at room temperature without microwave radiation. This observation indicates that the improvement of reaction kinetics was not due to a variation of the swelling properties of the polymer upon microwave heating.

The physical stability of PEGA_1900_ polymer to microwave treatment was verified by analyzing samples of beads after microwaves irradiation (Figure 4). After 1 hour of microwave radiation at 100°C in water no morphological modification of the beads was observed, and after 1 hour of irradiation in DMF at 150°C less than 1% of the beads (determined by beads counting) showed any evident damage in their structure (Figure 4(b)).

An indirect evaluation of the chemical stability of PEGA_1900_ was obtained by comparing the loading capacity of the resin before and after the irradiation. After 1 hour of treatment at 100°C under microwave irradiation it was verified that 0.145 mmol/g of functional groups were still accessible to Fmoc-Phe out of the initial 0.150 mmol/g. This was also confirmed by reacting the beads–after being irradiated with MW at 100°C—with dansyl chloride and analyzing them with two-photon-microscopy. A good uniformity of the fluorescence was measured (data not shown) demonstrating that MW treatment does not affect the reactivity.

A severe decrease of the loading capacity of the polymer was observed only after irradiation at 150°C that caused the loss of 90% of the initial accessible amino groups (0.150 mmol/g).

In conclusion, we here demonstrated that PEGA_1900_ is applicable to both chemical and enzymatic peptide synthesis under microwave irradiation. The benefit of MW radiation in terms of improvement of reaction kinetics has been demonstrated both in the chemical and enzymatic coupling. In the case of enzymatic coupling an optimum temperature of 80°C allows to achieve maximum reaction rates while avoiding the fast enzyme denaturation observable at higher temperature.

## Figures and Tables

**Scheme 1:**
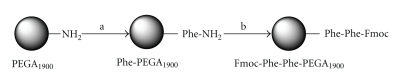
Synthesis of Fmoc-Phe-Phe-PEGA_1900_ under microwave radiation via enzymatic and chemical coupling. (a) (i) DIC/DMAP in DMF; (ii) Pyp 20%. (b) Chemical coupling (DIC/HOBt in DMF) or enzymatic coupling (Thermolysin in aqueous buffer) (see Supplementary Material available online at doi:10.1155/2009/362482).

**Figure 1:**
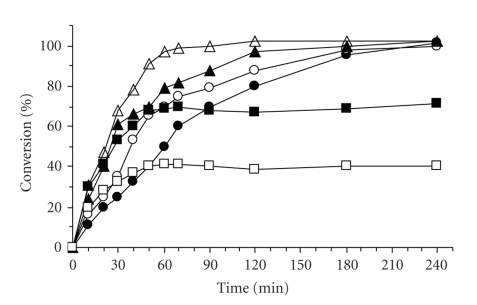
Reaction profiles of the enzymatic synthesis of Fmoc-Phe-Phe-PEGA_1900_, under microwave radiation at 50°C (bold circles), 60°C (empty circles), 70°C (bold triangles), 80°C (empty triangles), 90°C (bold squares), and 100°C (empty squares).

**Figure 2:**
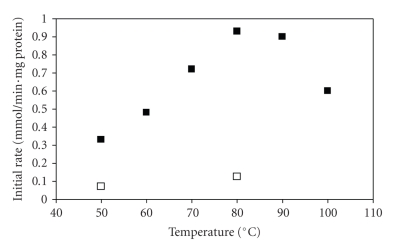
Initial rates of enzymatic reactions performed under microwave radiation (black squares) and without microwave radiation (empty squares) at different temperatures.

**Figure 3:**
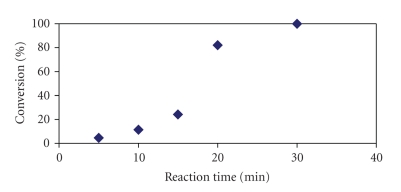
Chemical coupling of Fmoc-Phe on PEGA_1900_ under microwave radiation at 50°C and 50 W. Determination of conversion (see Supplementary Material).

**Figure 4:**
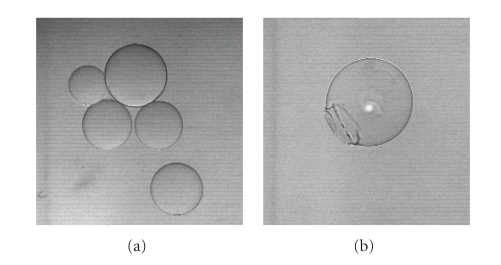
(a) Physical appearance of PEGA_1900_ beads after microwave radiation at 150°C in DMF; (b) a detail of the damaged beads counted in a Burker chamber (less than 1% of total).
